# Atypical Lower Limb Mechanics During Weight Acceptance of Stair Descent at Different Time Frames After Anterior Cruciate Ligament Reconstruction

**DOI:** 10.1177/03635465221095236

**Published:** 2022-05-23

**Authors:** Jonas L. Markström, Dario G. Liebermann, Lina Schelin, Charlotte K. Häger

**Affiliations:** † Department of Community Medicine and Rehabilitation, Physiotherapy, Umeå University, Umeå, Sweden; ‡ Department of Statistics, Umeå School of Business, Economics and Statistics, Umeå University, Umeå, Sweden; § Department of Physical Therapy, Stanley Steyer School of Health Professions, Sackler Faculty of Medicine, Tel Aviv University, Tel Aviv, Israel; Investigation performed at Umeå University, Umeå, Sweden

**Keywords:** ACL, stepping down, motion analysis, biomechanics, functional data analysis

## Abstract

**Background:**

An anterior cruciate ligament (ACL) rupture may result in poor sensorimotor knee control and, consequentially, adapted movement strategies to help maintain knee stability. Whether patients display atypical lower limb mechanics during weight acceptance of stair descent at different time frames after ACL reconstruction (ACLR) is unknown.

**Purpose:**

To compare the presence of atypical lower limb mechanics during the weight acceptance phase of stair descent among athletes at early, middle, and late time frames after unilateral ACLR.

**Study Design:**

Controlled laboratory study.

**Methods:**

A total of 49 athletes with ACLR were classified into 3 groups according to time after ACLR—early (<6 months; n = 17), middle (6-18 months; n = 16), and late (>18 months; n = 16)—and compared with asymptomatic athletes (control; n = 18). Sagittal plane hip, knee, and ankle angles; angular velocities; moments; and powers were compared between the ACLR groups’ injured and noninjured legs and the control group as well as between legs within groups using functional data analysis methods.

**Results:**

All 3 ACLR groups showed greater knee flexion angles and moments than the control group for injured and noninjured legs. For the other outcomes, the early group had, compared with the control group, less hip power absorption, more knee power absorption, lower ankle plantarflexion angle, lower ankle dorsiflexion moment, and less ankle power absorption for the injured leg and more knee power absorption and higher vertical ground reaction force for the noninjured leg. In addition, the late group showed differences from the control group for the injured leg revealing more knee power absorption and lower ankle plantarflexion angle. Only the early group took a longer time than the control group to complete weight acceptance and demonstrated asymmetry for multiple outcomes.

**Conclusion:**

Athletes with different time frames after ACLR revealed atypically large knee angles and moments during weight acceptance of stair descent for both the injured and the noninjured legs. These findings may express a chronically adapted strategy to increase knee control. In contrast, atypical hip and ankle mechanics seem restricted to an early time frame after ACLR.

**Clinical Relevance:**

Rehabilitation after ACLR should include early training in controlling weight acceptance. Including a control group is essential when evaluating movement patterns after ACLR because both legs may be affected.

A rupture of the anterior cruciate ligament (ACL) is a common knee injury during sports^52^ that, despite rehabilitation protocols with or without ACL reconstruction (ACLR), still results in numerous short- and long-term consequences. These consequences include lower self-rated knee function and quality of life,^19,34,54^ fear of movement,^3,5^ decreased sports participation,^6,20^ increased weight gain,^37^ knee osteoarthritis,^10^ and reduced functional performance outcomes.^24,29,32,45,54^ In addition, an ACL injury results in a loss of mechanically sensitive receptors initially found in the ruptured ACL.^2,47^ This loss is hypothesized to lead to changes in afferent information to the central nervous system, resulting in disturbed reactive neuromuscular control of the knee joint and, consequently, adapted movement strategies for compensation.^38,39^ Therefore, patients with ACLR may present atypical lower limb mechanics as a strategy to maintain knee stability during lower limb loading, irrespective of time after ACLR, possibly resulting in harmful long-term consequences.

The presence of atypical lower limb mechanics at different times after unilateral ACLR has been evaluated during gait,^14,18,22,25^ jogging,^22^ and hop for distance.^40^ Although the times after ACLR differed between these studies, ranging from weeks to years, a common finding was that the injured leg, and in some instances, the contralateral noninjured leg also, displayed a knee loading avoidance pattern compared with asymptomatic controls.^14,18,22,25,40^ The movement patterns became less atypical with time but, in most cases, did not fully normalize to resemble controls or the contralateral leg.^14,22,25,40^ Further studies with a longitudinal study design or multiple groups of patients with different times after ACLR are needed to verify these findings.

Stair descent is a suitable task for evaluating lower limb mechanics at different times after ACLR; it constitutes an essential aspect of knee function and is more demanding than other tasks encountered in everyday life, including gait, chair sit-to-stand, and squat.^30,36^ The weight acceptance phase of stair descent requires lower limb control to stabilize the limb and absorb shock while undergoing sagittal plane knee joint excursion during the relatively slow execution of the task.^35^ Multiple studies have evaluated the presence of atypical lower limb mechanics among patients with ACLR during stair descent but present diverse findings.^9,21,26,31,46,50,57^ Some report different knee mechanics between the ACLR group and controls,^21,26,31,46,57^ while others found no differences.^9,50^ These studies evaluated different outcomes and parts of the stair descent gait cycle among patients with times after ACLR ranging from a few months to >20 years, making it difficult to generalize these findings. However, no study that we are aware of has performed repeated testing over time or tested patients with different times after ACLR while focusing on the weight acceptance phase. In addition, all but 1 of the stair descent studies^50^ mentioned above reduced the kinematic and kinetic curve (time series) data to discrete outcomes extracted during a predefined phase (eg, peak angle and moment). While the analysis of discrete outcomes is standard practice, it may exclude valuable information provided by inferential methods analyzing curve data.^42,55^

In this study, we aimed to evaluate and compare the presence of atypical lower limb mechanics during the weight acceptance phase of stair descent among athletes at early, middle, and late time frames after unilateral ACLR using statistical methods from functional data analysis. This information is necessary to better understand the consequences of ACLR to improve the elements of rehabilitation targeting knee joint motor control for these patients. We hypothesized that all ACLR groups would display a hip and knee flexion strategy with increased knee force absorption over a longer time of the weight acceptance phase to cope with poorer sensorimotor knee control. We also hypothesized that between-leg asymmetry would be evident early after ACLR and still evident but less pronounced among the middle and late groups.

## Methods

### Study Design

This was a controlled laboratory study approved by the regional ethical review board in Umeå, Sweden (Dnr. 2015/67-31). Before partaking in the study, all patients provided written informed consent and participated in agreement with the Declaration of Helsinki. The participants were tested at U-Motion Laboratory.

### Participants

This study included 18 asymptomatic athletes (control; 15 female; mean age, 21.8 ± 2.7 years; mean body mass index, 22.4 ± 2.1) and 49 athletes with ACLR (29 female; mean age, 24.4 ± 4.7 years; mean body mass index, 23.6 ± 2.4) who were stratified into groups based on time after ACLR: early (<6 months; mean, 2.9 ± 1.1 months after ACLR; n = 17), middle (6-18 months; mean, 10.0 ± 2.1 months after ACLR; n = 16), and late (>18 months; mean, 52.3 ± 34.0 months after ACLR; n = 16). All participants in this study had a Tegner activity scale^53^ rating of at least 7 of 10 before injury. Patients with ACLR suffered their ACL injury during sports participation (35 noncontact, 8 indirect contact, 6 contact; classified from an interview) and had reconstruction with an ipsilateral hamstring tendon autograft, as is national standard practice. They were recruited from the orthopaedic clinic of the regional hospital by the same knee-specialized physical therapist and, in a few cases, from a local sports medicine clinic and advertisements around the university and hospital campus. They met the following inclusion criteria: 17-34 years of age, unilateral ACL injury, no complete tear of any other knee ligament, no major meniscal or articular damage, no severe ankle sprain in the past 6 months, and no other musculoskeletal/neurological abnormality that would affect test performance. Before testing, all participants had to be able to descend and ascend stairs without any aid and to be able to stand up and sit down again from a chair by only using their injured leg. These criteria were checked via a telephone interview before testing. Participants in the control group competed in the highest or second highest national league in floorball or soccer and were recruited from sports clubs. They met the same relevant inclusion criteria and underwent a clinical knee examination by an experienced physical therapist in addition to a telephone interview for screening before testing.

### Experimental Task: Stair Descent

Participants performed the stair descent task after application of a marker setup and collection of stationary recording data while standing for modeling purposes. The task included 2 steps set at a relative height of 22% and 11% of the participant's height, based on the lowest expected body height of approximately 1.5 to 1.6 m that relates to a standard stair step height of 17 cm. Body height–normalized stair step heights are appropriate for interindividual comparisons of joint biomechanics because they influence lower limb joint angles, moments, and powers.^51^ The test was performed with the participant barefoot at a self-selected speed while holding a 25-cm rope with both hands behind the back to standardize arm movements, avoid hiding relevant markers that generated the model, and emphasize lower limb control. Participants were instructed to step down as naturally as possible and continue walking forward for 3 to 4 additional steps when on ground level. They had 1 familiarization trial per leg and then completed 6 trials for each leg, alternating the leading leg for every trial. The same test leader (J.L.M.) gave instructions, palpated, and controlled for marker placement for all but 5 test sessions, where data were collected by another trained test leader using the same protocol.

### Instruments and Data Processing

Three-dimensional ground-reaction force (GRF) data were collected at 1200 Hz using a force plate (9260AA6; Kistler) during the first step. These data were filtered at 50 Hz and synchronized with 3-dimensional kinematic data collected at 240 Hz using a system with 8 Oqus optoelectronic cameras (Qualisys). A 15-segment 6 degrees of freedom model was constructed in Visual3D (Version 5.02.30; C-Motion) from 56 passive spherical markers attached with double-coated adhesive tape on the skin at anatomic landmarks, as previously described in detail.^33^ Participants wore rigid clusters with 4 markers on each thigh to improve construct validity by reducing the effects of soft tissue artifacts.^12^ The marker data were tracked with Qualisys Track Manager software (Version 2.11) and filtered at 15 Hz with a critically dampened digital filter before being used to calculate the outcome variables. A functional joint method was used to define hip joint centers from hip circumduction movement with the pelvis as a reference.^48^ Knee and ankle joint centers were defined as the midpoint between markers on the femoral epicondyles and malleoli, respectively.

Joint kinematics was calculated using the Cardan rotation sequence *XYZ* (*X*, mediolateral axis; *Y*, anteroposterior axis; *Z*, longitudinal axis)^11^ and determined from the movement of the distal segment relative to the proximal segment. Joint kinetics was calculated with inverse dynamics using a resultant moment approach,^7^ with coordinate systems determined in the proximal segment coordinate system. Moments were normalized to body weight and expressed as external moments. The segments’ masses were calculated based on the proportions of Dempster.^17^ Kinematic and kinetic data were filtered at 15 Hz with a fourth-order bidirectional zero-lag low-pass Butterworth digital filter.

### Outcome Variables

Kinematic and kinetic data were analyzed during weight acceptance, defined between initial contact for the leading leg on the force plate (vertical force >20 N) to toe-off of the lagging leg (toe marker horizontal speed >5% of maximum during the trial). The primary outcomes were time-series (curve) data for sagittal plane hip, knee, and ankle joint angles; angular velocities; moments; and powers during weight acceptance. Analyses were restricted to the sagittal plane because lower limb movement primarily occurs in this plane during stair descent, mainly being the plane in which adopted movement strategies to increase knee control would be revealed. In addition, curve data for the body weight–normalized vertical GRF were also analyzed. A secondary outcome was the time to complete the weight acceptance phase because time may easily be manipulated as a strategy to control the rate of vertical GRF increase due to Newton's second law (impulse-momentum relationship).

### Statistical Analysis

Comparisons were made between the 3 ACLR groups’ injured leg and the control nondominant leg (nonpreferred leg to kick a ball), the ACLR groups’ noninjured leg and the control dominant leg, and between the legs within each group. The kinematic and kinetic mean curves were analyzed with statistical methods of functional data analysis. The weight acceptance phase was discretized into 101 points, and individual mean curves were calculated across trials for each outcome variable. These time-normalized mean curves were used for statistical comparisons between groups and between legs within groups. The kinematic and kinetic curves were analyzed and compared between the ACLR groups (early, middle, late) and the control group by applying a functional linear model based on the interval-wise testing procedure.^1^ The following model was used to describe each kinematic and kinetic curve, $y_{i}\left(t\right)$ :

$$\begin{array}{c} y_{i}\left(t\right)=\beta_{0}\left(t\right)+\beta_{EARLY}\left(t\right)x_{EARLY,i}+\beta_{MID}\left(t\right)x_{MID,i}\\ +\beta_{LATE}\left(t\right)x_{LATE,i}+\varepsilon_{i}\left(t\right),i=1,\ldots .,67,t\in [0,100], \end{array}$$

where $x_{EARLY,i}$ , $x_{MID,i}$ , and $x_{LATE,i}$ are indicator functions attaining the value of 1 if participant i is included in the early, middle, or late group, respectively, and otherwise 0, with the control group used as a reference. First, a global overall significance test was performed that simultaneously examined if at least 1 of the coefficients (early, middle, late) was statistically significant (differed relative to the control) somewhere in the domain. Significant results were followed with separate post hoc tests for each coefficient to analyze how each ACLR group differed from the control group. For between-leg comparisons within each group, functional paired *t* tests based on the interval-wise testing procedure were used.^43^ Interval-wise testing–adjusted *P* values were used to evaluate statistical significance for all comparisons. Significant results of domains smaller than 5% of the phase were not interpreted for all functional analyses. Finally, the time to complete the weight acceptance phase was analyzed using analysis of variance with planned contrasts between the ACLR groups and the control group and using paired *t* tests between legs within groups for symmetry. All computations and statistical analyses of curve data were conducted using R (Version 3.6.1), while statistical analyses of discrete data were performed with SPSS (Version 25; IBM). A 5% level for statistical significance was set a priori.

## Results

### Between-Group Differences for Injured Leg

**Figure 1.**
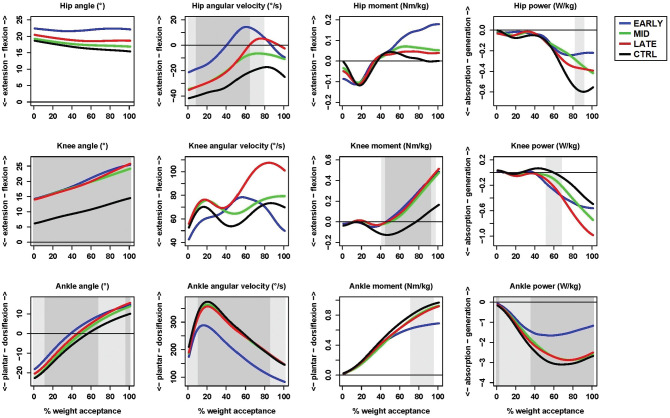
Group mean hip, knee, and ankle kinematic and kinetic curves for the anterior cruciate ligament reconstruction (ACLR) groups’ injured leg compared with the control (CTRL) nondominant leg. The gray areas within the plots indicate significant differences between any of the ACLR groups and the control group with adjusted *P* < .05 (light gray) and *P* < .01 (dark gray).

The functional linear model's overall test revealed significant differences (adjusted *P* < .05) between the ACLR groups and the control group at the hip, knee, and ankle during various periods of the time-normalized weight acceptance phase (Figure 1; see Appendix 1, available in the online version of this article, for group mean curves together with individual mean curves to see how data vary and *P* value curves for the injured leg during weight acceptance).

The post hoc tests revealed the following: the early group had lower hip extension angular velocity, less hip power absorption, higher knee flexion angle, higher knee external flexion moment, greater knee power absorption, lower ankle plantarflexion angle, lower ankle dorsiflexion angular velocity, lower ankle external dorsiflexion moment, and less ankle power absorption than the control group; the middle group had higher knee flexion angle and higher knee external flexion moment than the control group; and the late group had higher knee flexion angle, higher knee external flexion moment, greater knee power absorption, and lower ankle plantarflexion angle than the control group (Table 1).

**Table 1:** Domains of Weight Acceptance (0%-100%) in Which Outcomes Significantly Differed Between 3 ACLR Groups and Control Group^*a*^

	Early vs Control		Middle vs Control		Late vs Control		
	Domain, %	Difference	Domain, %	Difference	Domain, %	Difference
Injured leg						
Hip extension angular velocity	0-82	20-38 deg/s less	—	—	—	—
Hip power absorption	77-99	0.25-0.38 W/kg less	—	—	—	—
Knee flexion angle	0-100	8.1-11.1 deg more	0-100	7.9-9.6 deg more	0-100	7.9-11.3 deg more
Knee flexion moment	42-97	0.10-0.31 Nċm/kg more	40-94	0.09-0.29 Nċm/kg more	38-98	0.08-0.34 Nċm/kg more
Knee power absorption	49-58	0.19-0.26 W/kg more	—	—	46-90	0.12-0.49 W/kg more
Ankle plantarflexion angle	0-100	4.6-6.4 deg less	—	—	59-97	4.1-5.5 deg less
Ankle dorsiflexion angular velocity	0-100	36-95 deg/s less	—	—	—	—
Ankle dorsiflexion moment	63-97	0.14-0.27 Nċm/kg less	—	—	—	—
Ankle power absorption	0-100	0.09-1.59 W/kg less	—	—	—	—
Noninjured leg						
Knee flexion angle	0-100	5.4-10.1 deg more	0-100	8.1-9.6 deg more	0-100	8.9-11.2 deg more
Knee flexion moment	27-99	0.09-0.42 Nċm/kg more	22-97	0.07-0.30 Nċm/kg more	22-100	0.06-0.39 Nċm/kg more
Knee power absorption	35-100	0.24-0.59 W/kg more	—	—	—	—
Vertical ground-reaction force	25-95	0.94-2.17 N/kg more	—	—	—	—

a “Difference” refers to the group mean differences between the early, middle, and late groups and the control group throughout the domain. Dashes indicate no statistical differences between the groups. Moments are described as external. Adjusted *P* < .05. ACLR, anterior cruciate ligament reconstruction.

The early group took a longer time to complete the weight acceptance phase than the control group (95% CI, 0.04-0.09 seconds longer [50% longer on average]; *P* < .001), while the middle and late groups had similar times to the control group (*P*≥ .292).

### Between-Group Differences for Noninjured Leg

**Figure 2.**
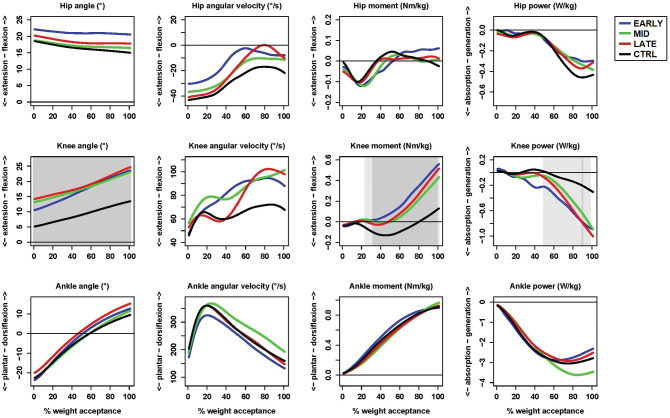
Group mean hip, knee, and ankle kinematic and kinetic curves for the anterior cruciate ligament reconstruction (ACLR) groups’ noninjured leg compared with the control (CTRL) dominant leg. The gray areas within the plots indicate significant differences between any of the ACLR groups and the control group with adjusted *P* < .05 (light gray) and *P* < .01 (dark gray).

The functional linear model's overall test revealed significant differences (adjusted *P* < .05) between the ACLR groups and the control group at the knee and for vertical GRF during various periods of the time-normalized weight acceptance phase (Figure 2; see Appendix 2, available in the online version of this article, for group mean curves together with individual mean curves to see how data vary and *P* value curves for the noninjured leg during weight acceptance).

The post hoc tests revealed the following: the early group had higher knee flexion angle, higher knee external flexion moment, greater knee power absorption, and higher vertical GRF than the control group; the middle group had higher knee flexion angle and higher knee external flexion moment than the control group; and the late group had higher knee flexion angle and higher knee external flexion moment than the control group (Table 1).

The early group took a longer time to complete the weight acceptance phase than the control group (95% CI, 0.01-0.06 seconds longer [26% longer on average]; *P* = .022), while the middle and late groups had similar times to the control group (*P*≥ .413).

### Between-Leg Differences Within Groups

The early and middle groups demonstrated asymmetry (adjusted *P* < .05) for hip and ankle mechanics but not knee mechanics. The late and control groups did not display asymmetry for any outcome. For the early group, the injured leg had lower hip extension angular velocity, higher hip external flexion moment, lower ankle plantarflexion angle, lower ankle dorsiflexion angular velocity, lower ankle external dorsiflexion moment, less ankle power absorption, and lower vertical GRF than the noninjured leg. For the middle group, the injured leg displayed lower ankle dorsiflexion angular velocity and less ankle power absorption than the noninjured leg (Table 2).

**Table 2:** Domains of Weight Acceptance (0%-100%) in Which Outcomes Significantly Differed Between Legs Within Groups^*a*^

	Early		Middle		Late	Control
	Domain, %	Difference	Domain, %	Difference	Domain, %	Domain
Hip extension angular velocity	10-33, 63-69	11-17 deg/s less	—	—	—	—
Hip flexion moment	67-100	0.08-0.12 Nċm/kg more	—	—	—	—
Ankle plantarflexion angle	0-91	2.3-5.7 deg less	—	—	—	—
Ankle dorsiflexion angular velocity	29-97	51-67 deg/s less	54-95	36-52 deg/s less	—	—
Ankle dorsiflexion moment	0-100	0.01-0.21 Nċm/kg less	—	—	—	—
Ankle power absorption	0-100	0.07-1.28 W/kg less	79-95	0.03-0.85 W/kg less	—	—
Vertical ground-reaction force	3-100	0.09-1.47 N/kg less	—	—	—	—

a “Difference” refers to the group mean differences between the injured leg and the noninjured leg throughout the domain. Dashes indicate no statistical differences between legs within groups. Moments are described as external. Adjusted *P* < .05.

A longer time to complete the weight acceptance phase for the injured leg was observed for the early group (95% CI, 0.01-0.05 seconds longer [16% longer on average]; *P* = .014) and middle group (95% CI, 0.01-0.04 seconds longer [19% longer on average]; *P* = .001), while the late and control groups had similar times for both legs (*P* = .481 and .517, respectively).

## Discussion

Our results confirmed our hypothesis for knee mechanics in which the early, middle, and late groups showed higher knee flexion angles and moments than the control group. In addition, the group mean angle and moment curves were almost identical among the ACLR groups. As expected, the early group had more atypical lower limb mechanics for the injured leg than did the middle and late groups; they stepped down more carefully than the control group with a 50% longer time to complete the weight acceptance phase, accompanied by atypical hip mechanics (lower extension angular velocity and power absorption) and ankle mechanics (lower plantarflexion angle, dorsiflexion angular velocity, dorsiflexion moment, and power absorption). In addition, the early group displayed asymmetric hip and ankle mechanics not found to the same extent among the other groups and took a longer time to complete weight acceptance for the injured leg compared with the noninjured leg. These asymmetric movement patterns at the hip and ankle were not evident among the middle and late groups to the same extent and, therefore, may normalize with time after ACLR.

Compared with the control group, the higher knee flexion angles and moments among the early, middle, and late groups may express an adapted movement strategy to increase knee control. This strategy may transform compressional tibiofemoral forces into moments that they can better control using the knee muscles. Anatomically, a greater knee flexion angle decreases the patellar tendon insertion angle and increases the hamstring tendon insertion angle relative to the tibial longitudinal axis in the sagittal plane,^15,27^ resulting in a better position to control for anterior tibial translation. A greater knee flexion angle also results in a lower posterior tibial slope, which is associated with less anterior tibial translation.^16,28^ In addition, a recent experimental study by Sharifi and Shirazi-Adl^49^ supports using a knee flexion strategy to increase knee stability after ACL injuries. Using a kinematics-driven musculoskeletal model of ACL-deficient knees, these authors showed that 2° to 6° greater knee flexion angles than reported by asymptomatic persons increased knee stability and decreased anterior tibial translation during the stance phase of gait.^49^ For comparison, the early, middle, and late groups had 8° to 11° greater knee flexion angles than the control group throughout weight acceptance for the injured leg (Table 1). All 3 ACLR groups also showed a knee extension moment avoidance pattern not found in the control group, accompanied with greater knee external flexion moments throughout the final approximate 60% period of weight acceptance than the control group. Interestingly, the contralateral noninjured leg among the early, middle, and late groups demonstrated similar knee mechanics to the injured leg, with 5° to 11° greater knee flexion angles, a knee extension moment avoidance pattern, and greater knee flexion moments throughout the final approximate 70% period of weight acceptance compared with the control group.

Neurophysiological consequences of an ACL injury may explain the atypical knee mechanics observed among all 3 ACLR groups involving both legs. The loss of mechanically sensitive receptors in the native ACL^2,47^ is hypothesized to lead to changes in afferent information to the central nervous system, resulting in disturbed reactive neuromuscular control of the knee joint and, consequently, to adapted movement strategies for compensation that may affect both limbs.^38,39^ A recent meta-analysis by Rodriguez et al^44^ strengthens this assertion, reporting bilateral reductions in the excitability of corticospinal pathways and voluntary activation of the quadriceps muscles, but bilaterally increased excitability of the spinal-reflex pathways (hypothesized to be a compensatory mechanism), among patients with ACLR compared with asymptomatic controls. In addition, patients with ACLR have shown different brain area activation with more engagement of visual-motor areas than sensory-motor areas compared with asymptomatic controls.^8,13,23^ As such, our results of atypical knee mechanics among the early, middle, and late groups may express altered motor representation involving both legs. In any case, considering that our findings and those of others^26,31,46,57^ show bilateral adaptations despite a unilateral ACL injury, the contralateral noninjured leg fails as a “normal” reference. Therefore, including a control group is essential when evaluating movement patterns after ACLR.

Our findings of atypical knee mechanics for all ACLR groups have clinical implications, suggesting that rehabilitation after ACLR should include early training in controlling weight acceptance because atypical knee mechanics does not seem to normalize with time. While most clinicians do not have a motion capture system at their disposal, or time to collect and process such data, simple video analysis using a smartphone and freely available apps may suffice to provide reliable data of knee flexion angles and contact time during stair descent.^41^ The clinician may use these data to quantify and evaluate movement control over time to improve the patient's knee control over the whole knee flexion-extension range. Indeed, neuromuscular knee control training is considered an essential component in ACL rehabilitation before returning to sports.^4^ Worse sensorimotor knee control is believed to increase the risk of positioning the knee joint in injury-prone positions during sports^38^ and, therefore, contributes to the heightened ACL reinjury risk.^56^ Our results suggest that early implementation of training to improve knee motor control may be beneficial. However, further research is needed to confirm our findings and evaluate their implications on future health after ACLR to better understand the consequences of ACLR on the capability to load the knee during stair descent.

This study has some strengths and limitations that need mentioning. Applying statistical methods for curve data (in our case, functional data analysis) is a strength because revealing results would not have been found if analyzing discrete values of peak and minimum values. A limitation is that we used a cross-sectional design to generalize lower limb mechanics for approximate stages of early, middle, and late time frames after ACLR. The cross-sectional design indicates that our results should not be used as evidence of how an atypical movement strategy persists or changes over time; longitudinal studies are needed to answer that question. Also, we used another test leader for 5 test sessions, which may have introduced intertester variability that affected the data. However, we did not observe any systematic differences in the motion curves when comparing the data collected from the 2 test leaders. Finally, we did not control the surgical procedure or rehabilitation after ACLR. However, all patients had an ipsilateral hamstring tendon autograft, and most participants were recruited from the orthopaedic clinic of the regional hospital by the same physical therapist responsible for their rehabilitation.

In conclusion, our findings show that patients with unilateral ACLR at early, middle, and late time frames after ACLR show similar and atypical knee joint mechanics with greater flexion angles and moments for both the injured and the noninjured legs during the weight acceptance phase of stair descent. These results indicate a chronically adapted movement strategy to maintain knee control when loading the leg. Patients early after ACLR also stepped down more carefully onto their injured leg using a longer time, accompanied with different ankle and hip mechanics than controls and the contralateral noninjured leg. We did not observe such findings to the same extent among patients with middle and late time frames after ACLR.

## Supplemental Material

sj-pdf-1-ajs-10.1177_03635465221095236 – Supplemental material for Atypical Lower Limb Mechanics During Weight Acceptance of Stair Descent at Different Time Frames After Anterior Cruciate Ligament Reconstruction Click here for additional data file. Supplemental material, sj-pdf-1-ajs-10.1177_03635465221095236 for Atypical Lower Limb Mechanics During Weight Acceptance of Stair Descent at Different Time Frames After Anterior Cruciate Ligament Reconstruction by Jonas L. Markström, Dario G. Liebermann, Lina Schelin and Charlotte K. Häger in The American Journal of Sports Medicine

sj-pdf-2-ajs-10.1177_03635465221095236 – Supplemental material for Atypical Lower Limb Mechanics During Weight Acceptance of Stair Descent at Different Time Frames After Anterior Cruciate Ligament Reconstruction Click here for additional data file. Supplemental material, sj-pdf-2-ajs-10.1177_03635465221095236 for Atypical Lower Limb Mechanics During Weight Acceptance of Stair Descent at Different Time Frames After Anterior Cruciate Ligament Reconstruction by Jonas L. Markström, Dario G. Liebermann, Lina Schelin and Charlotte K. Häger in The American Journal of Sports Medicine
