# Supplementary material for: Atypical Lower Limb Mechanics During Weight Acceptance of Stair Descent at Different Time Frames After Anterior Cruciate Ligament Reconstruction
Source: Am J Sports Med. 2022 May 23;50(8):2125–33. doi: 10.1177/03635465221095236 (PMC9227952; doi:10.1177/03635465221095236)
Supplement: sj-pdf-1-ajs-10.1177_03635465221095236 – Supplemental material for Atypical Lower Limb Mechanics During Weight Acceptance of Stair Descent at Different Time Frames After Anterior Cruciate Ligament Reconstruction [file sj-pdf-1-ajs-10.1177_03635465221095236.pdf]

Appendix 1. Results of the overall model comparing the three ACLR groups injured leg to CTRL non-dominant leg.

Figure descriptions for the following pages.

**Plot 1 (top)**			
**EARLY = blue**	**MID = green**	**LATE = red**	**CTRL = black**
Bold line = group grand mean curves		Light gray area = adjusted p value < .05	
Dotted lines = individual mean curves		Dark gray area = adjusted p value < .01	
Black dotted line = indicates the value of 0			
**Plot 2 (bottom)**			
Alpha .05 = red solid line		Unadjusted p values = gray line	
Alpha .01 = red dashed line		Adjusted p values = black line	

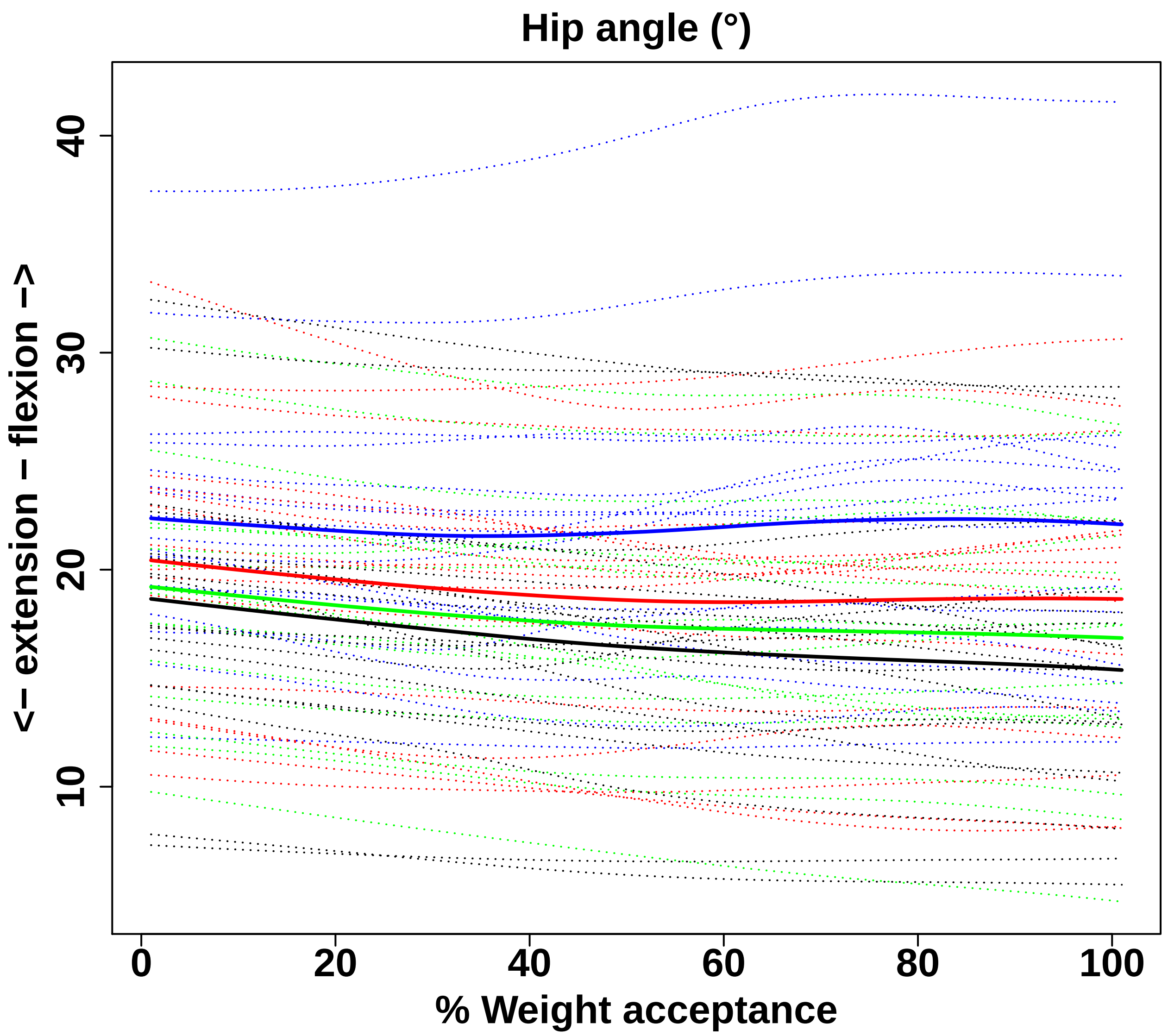

Hip angle (°)
<– extension – flexion –>
10
20
30
40
0
20
40
60
80
100
% Weight acceptance

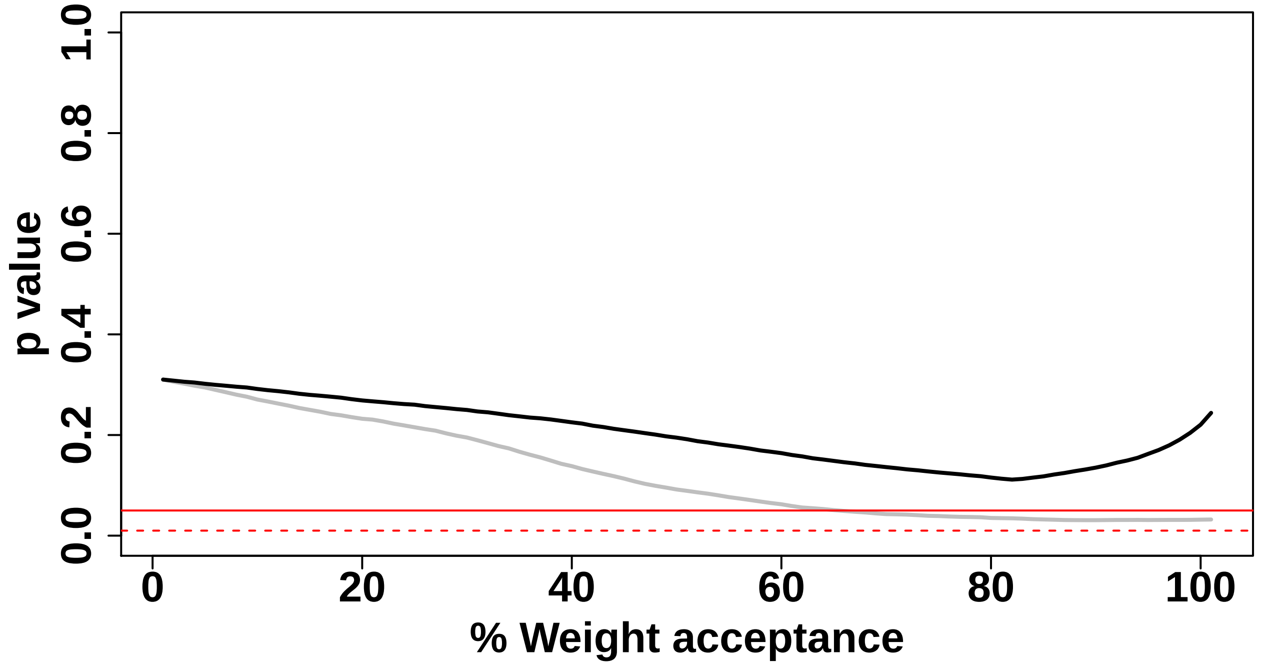

p value
0.0
0.2
0.4
0.6
0.8
1.0
0
20
40
60
80
100
% Weight acceptance

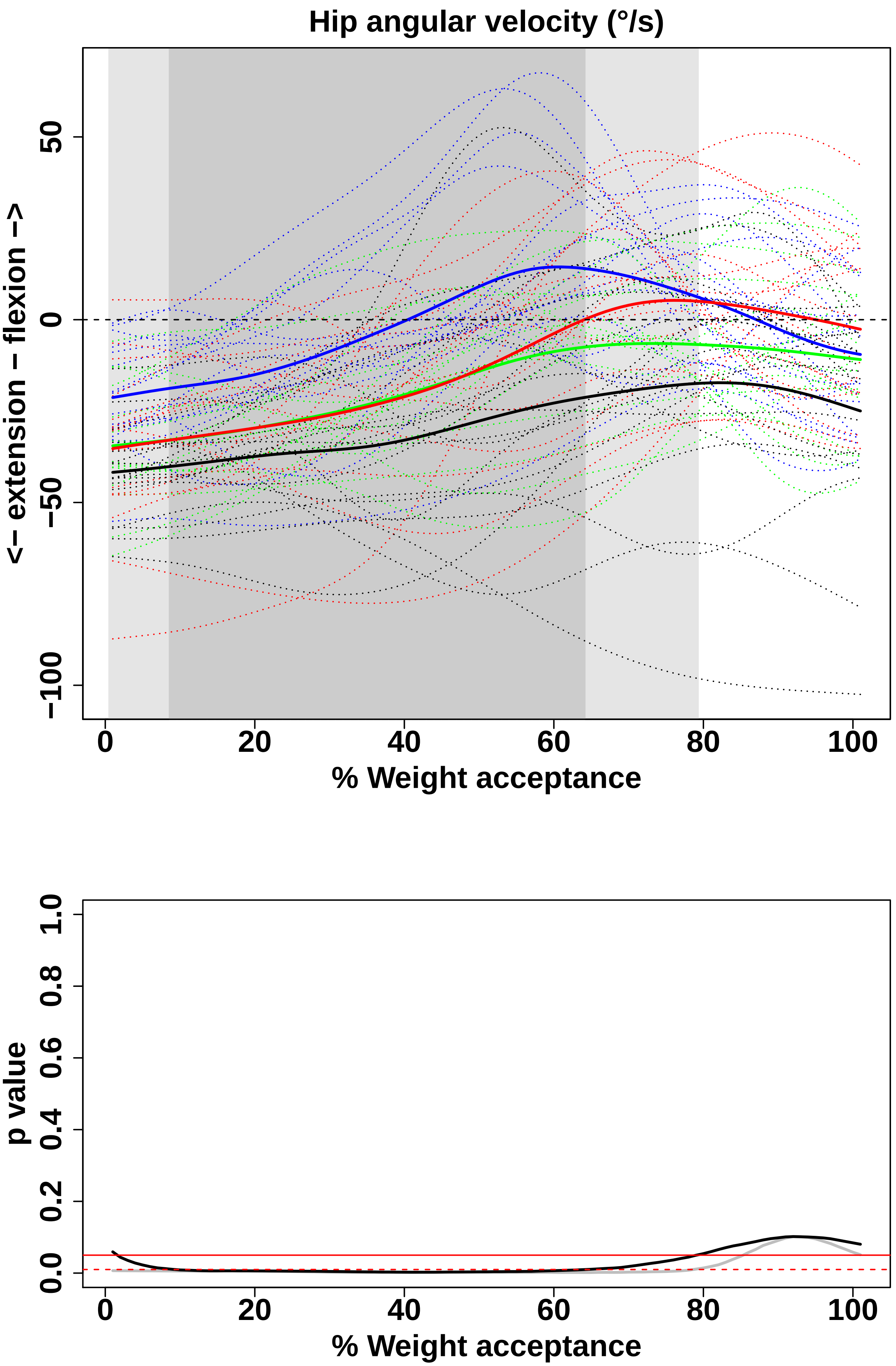
Hip angular velocity (°/s)
<– extension – flexion –>
50
0
−50
−100
0
20
40
60
80
100
% Weight acceptance
p value
0.0
0.2
0.4
0.6
0.8
1.0
0
20
40
60
80
100
% Weight acceptance

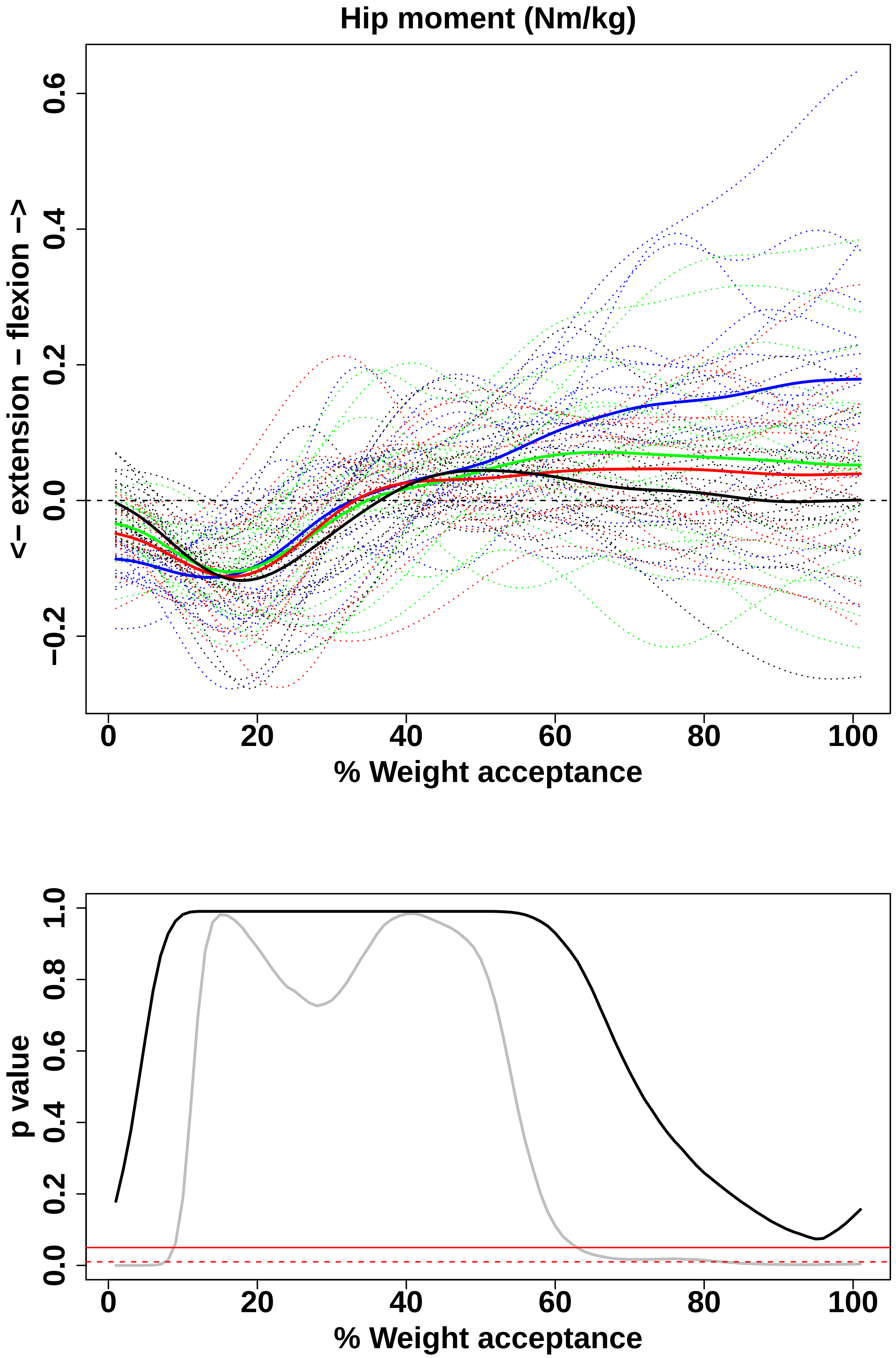

Hip moment (Nm/kg)
<– extension – flexion –>
0.6
0.4
0.2
0.0
−0.2
0
20
40
60
80
100
% Weight acceptance
p value
1.0
0.8
0.6
0.4
0.2
0.0
0
20
40
60
80
100
% Weight acceptance

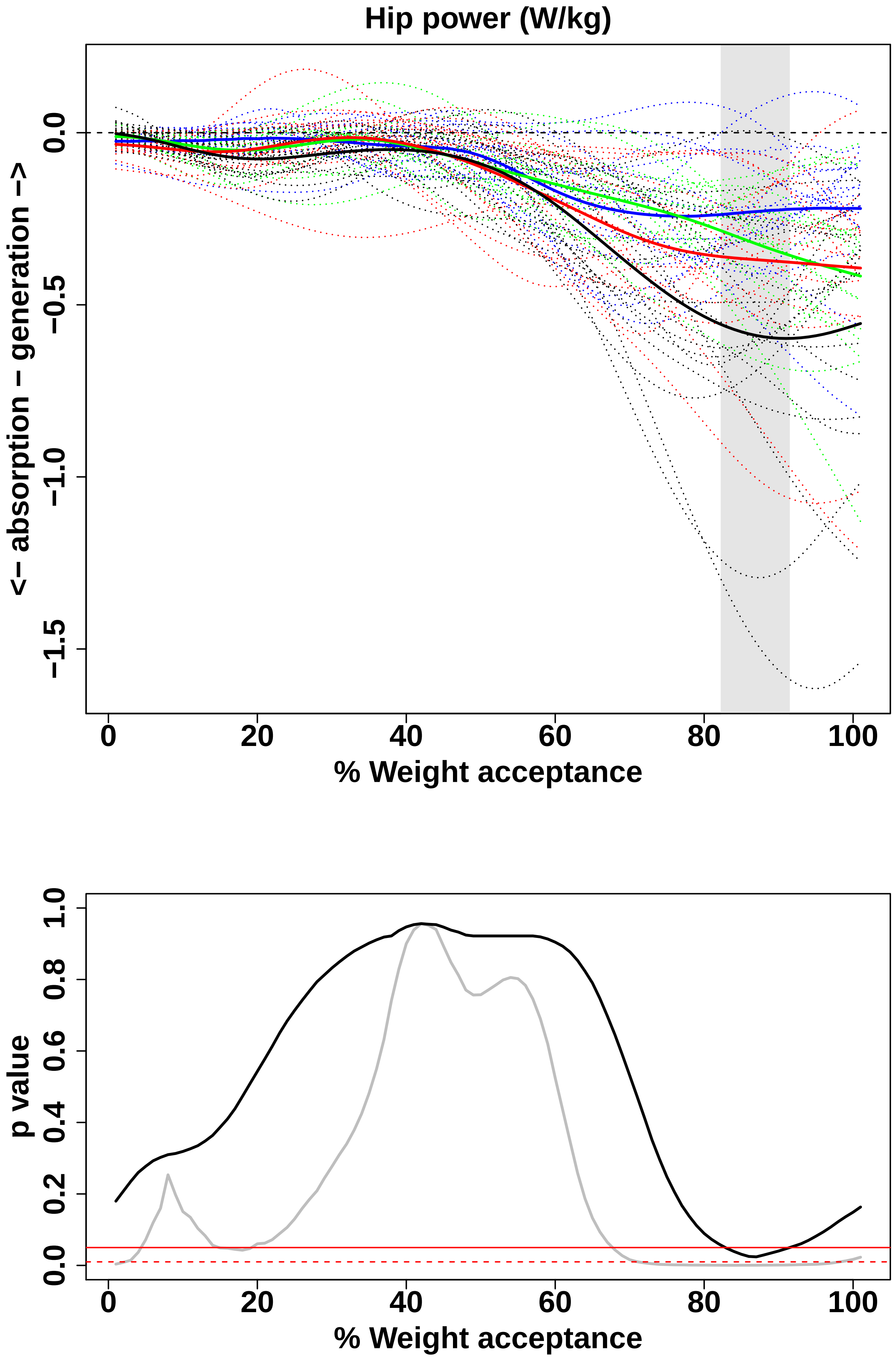
Hip power (W/kg)
<– absorption – generation –>
0.0
−0.5
−1.0
−1.5
0
20
40
60
80
100
% Weight acceptance
p value
0.0
0.2
0.4
0.6
0.8
1.0
0
20
40
60
80
100
% Weight acceptance

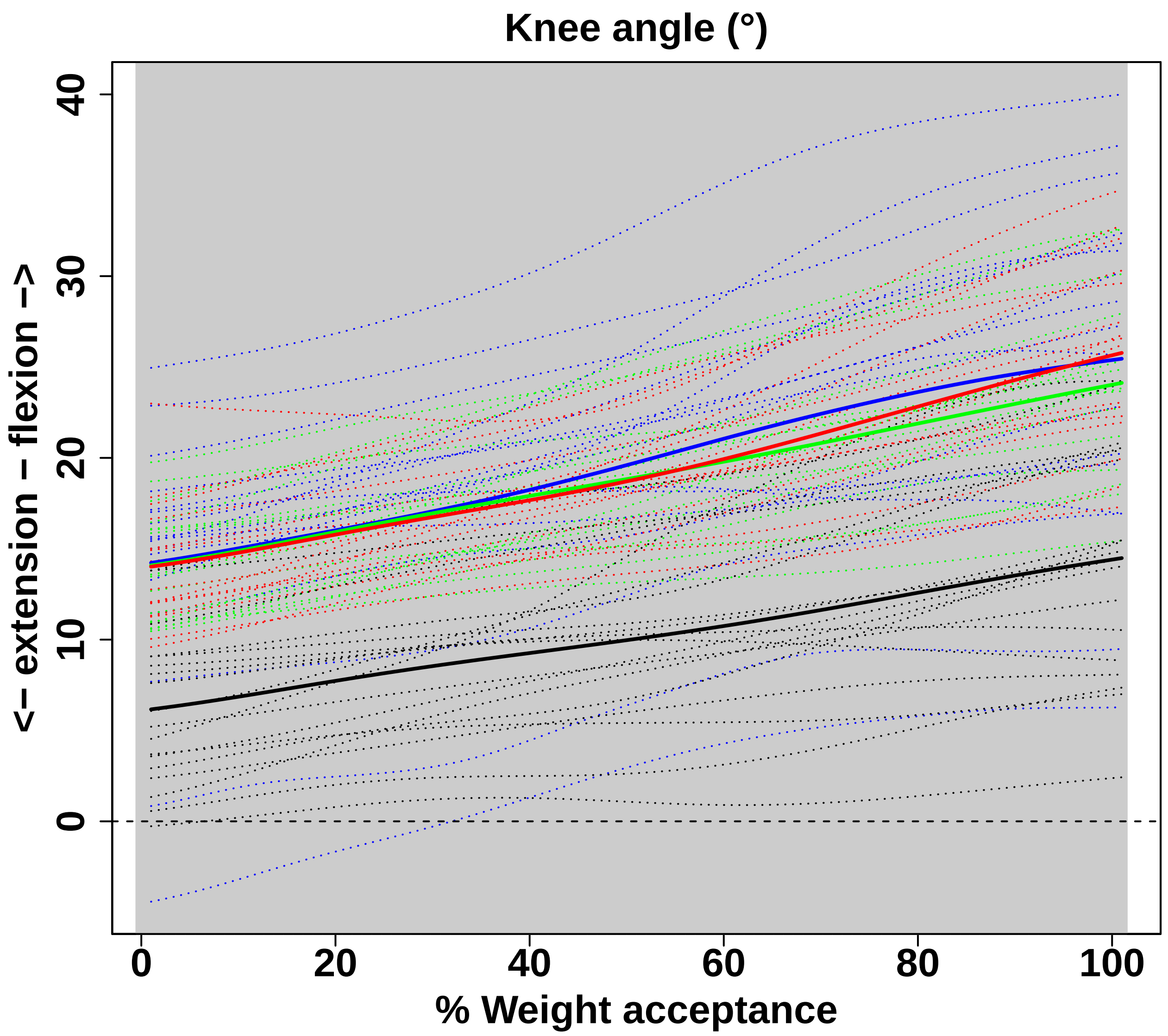
Knee angle (°)
<- extension – flexion ->
0
10
20
30
40
0
20
40
60
80
100
% Weight acceptance

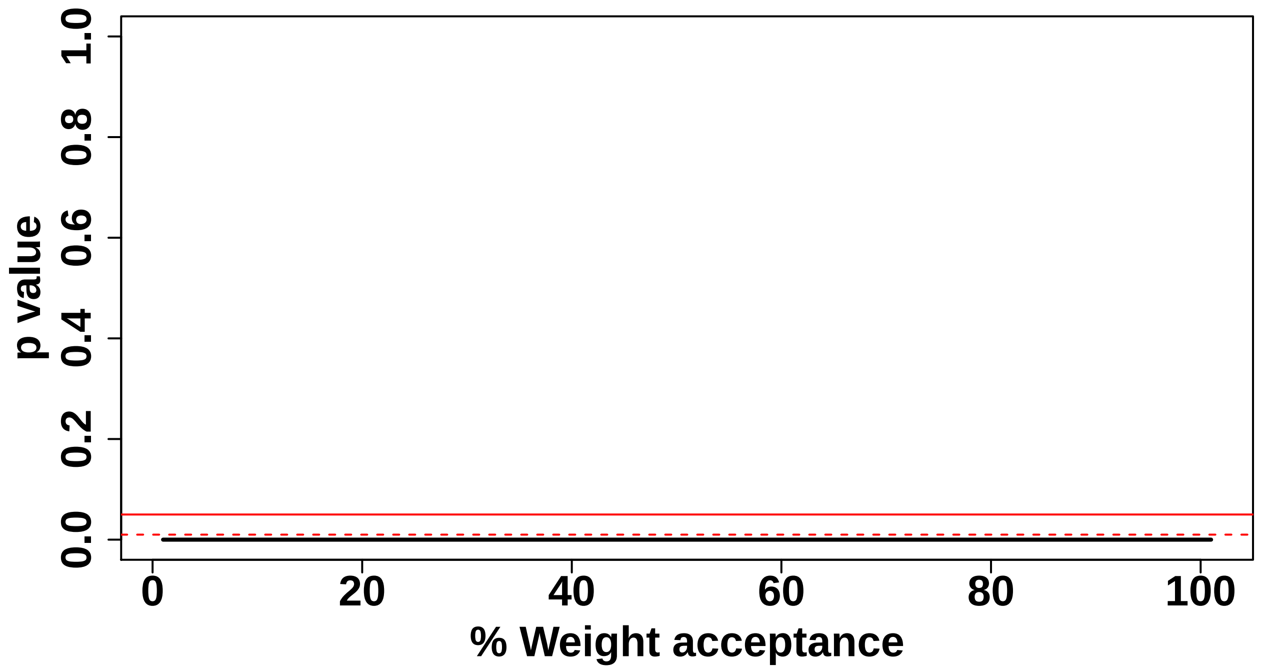
p value
0.0
0.2
0.4
0.6
0.8
1.0
0
20
40
60
80
100
% Weight acceptance

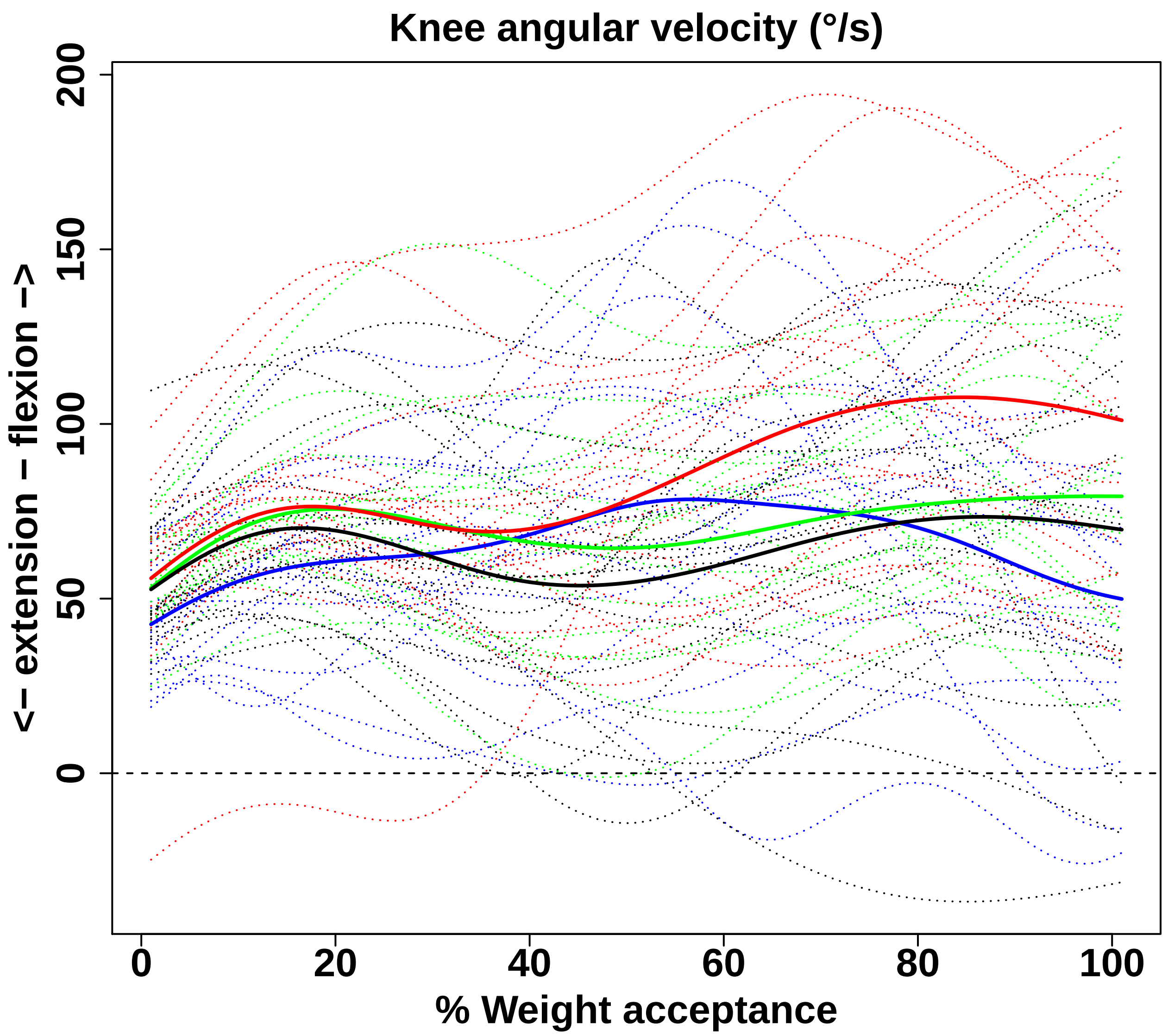
Knee angular velocity (°/s)
<– extension – flexion –>
0
50
100
150
200
0
20
40
60
80
100
% Weight acceptance

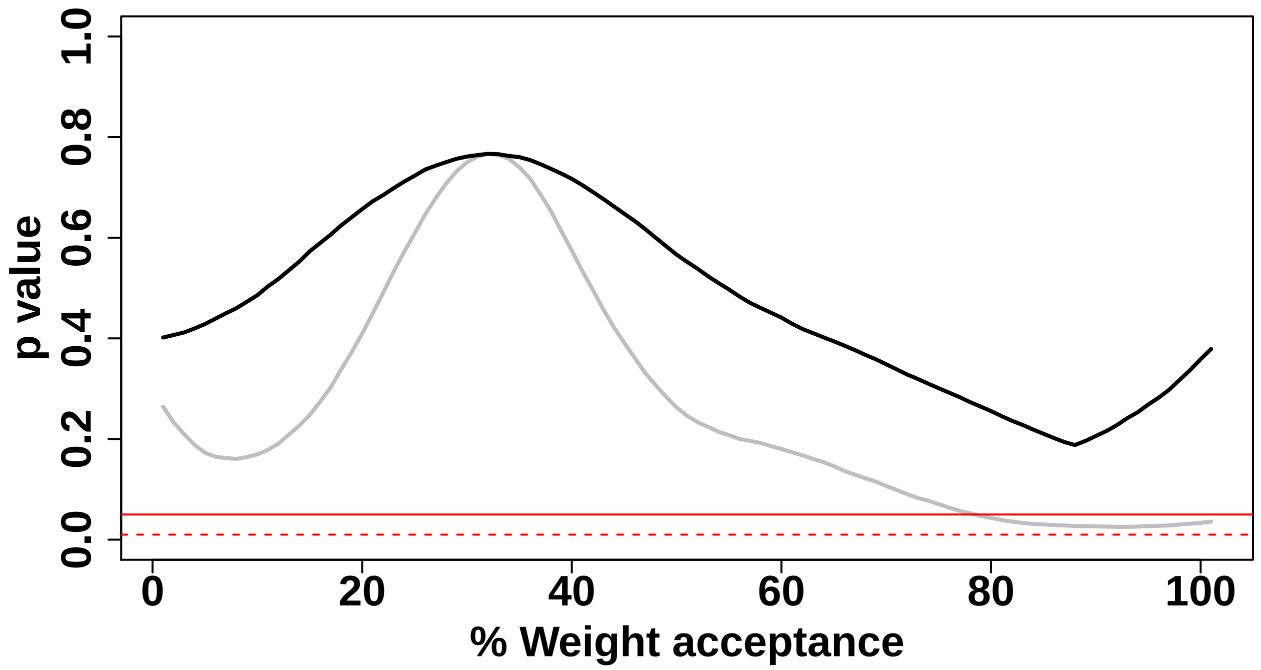
p value
0.0
0.2
0.4
0.6
0.8
1.0
0
20
40
60
80
100
% Weight acceptance

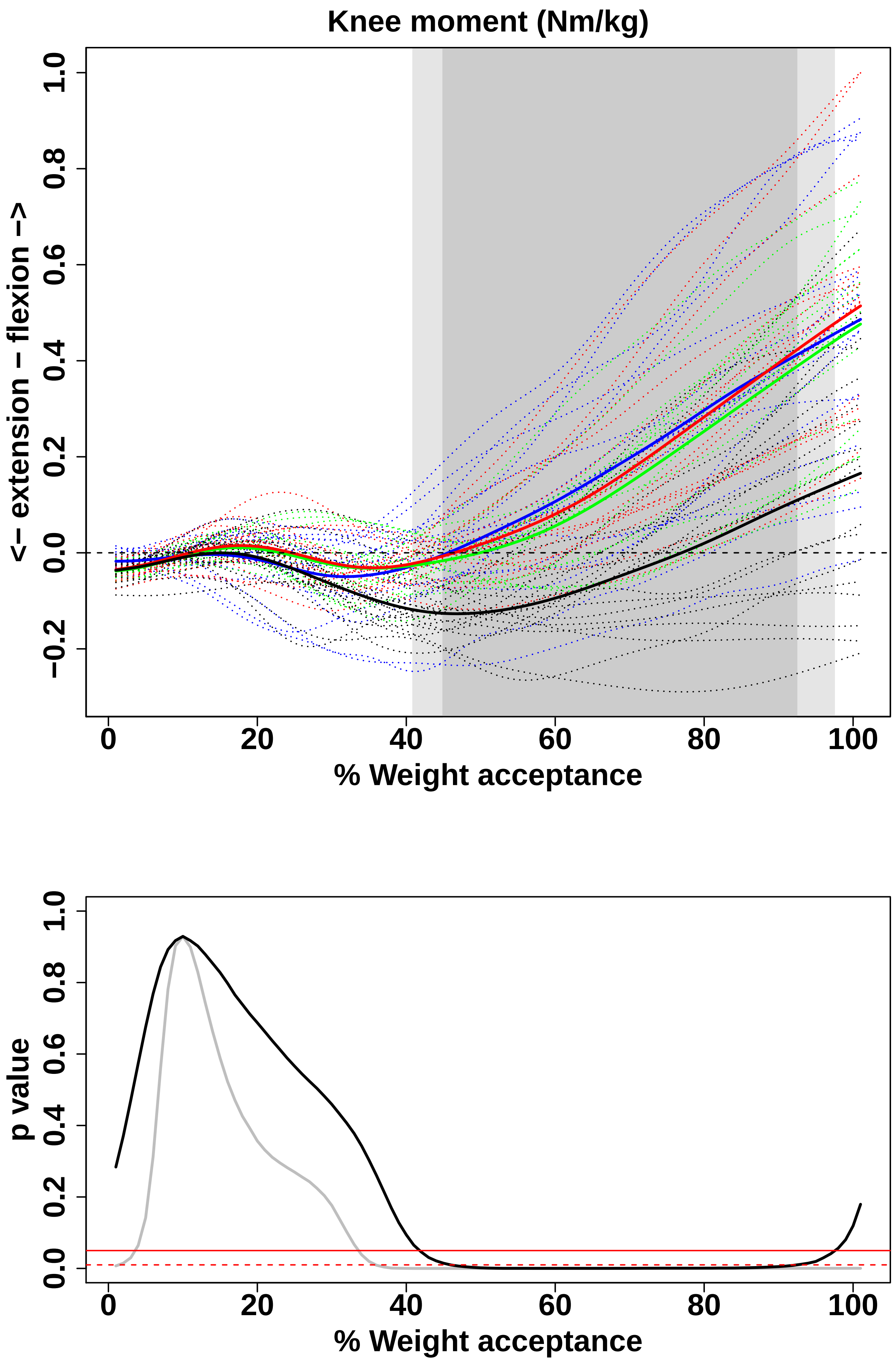
Knee moment (Nm/kg)
<– extension – flexion –>
1.0
0.8
0.6
0.4
0.2
0.0
−0.2
0
20
40
60
80
100
% Weight acceptance
p value
1.0
0.8
0.6
0.4
0.2
0.0
0
20
40
60
80
100
% Weight acceptance

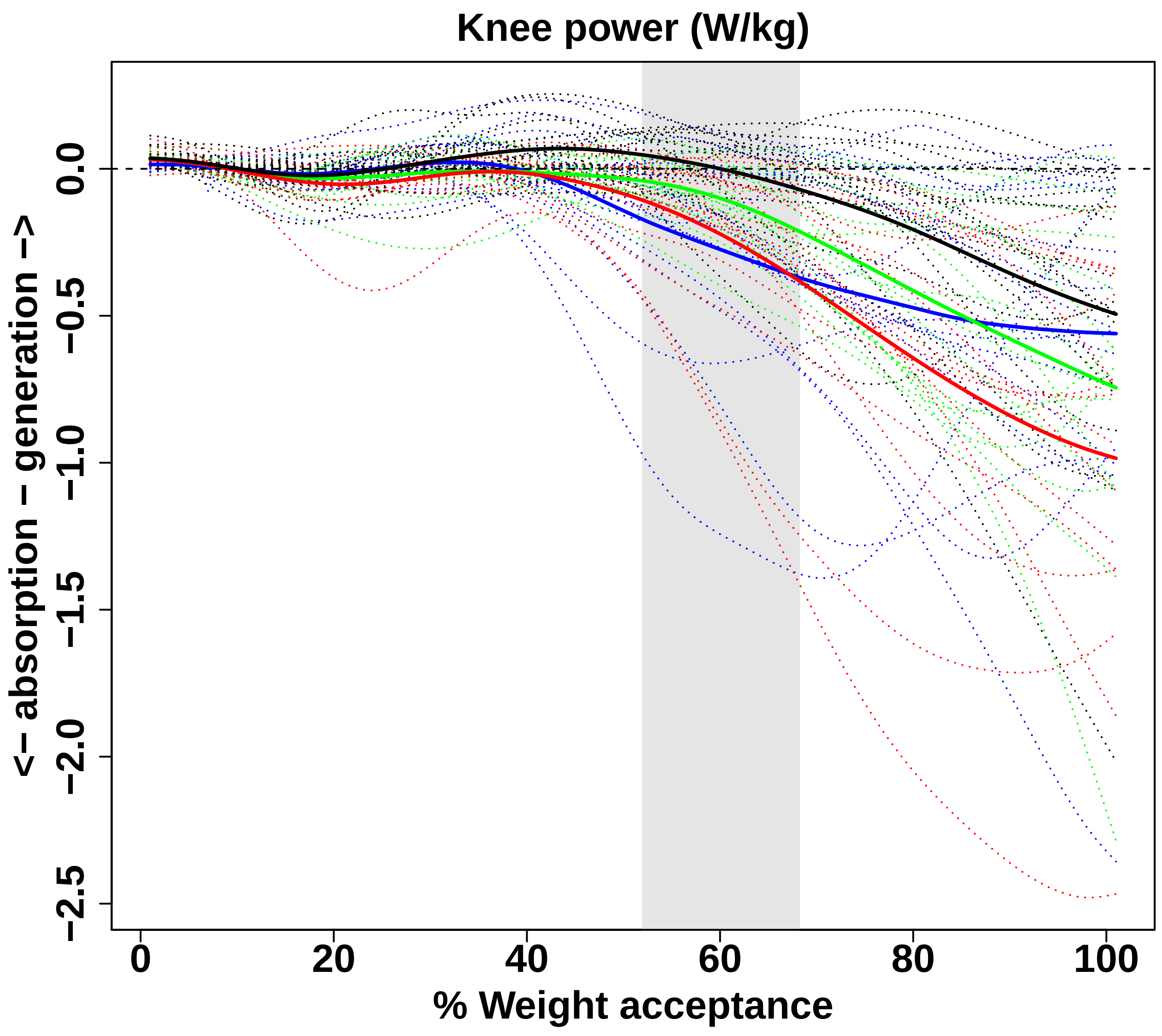

Knee power (W/kg)
<– absorption – generation –>
0.0
−0.5
−1.0
−1.5
−2.0
−2.5
0
20
40
60
80
100
% Weight acceptance

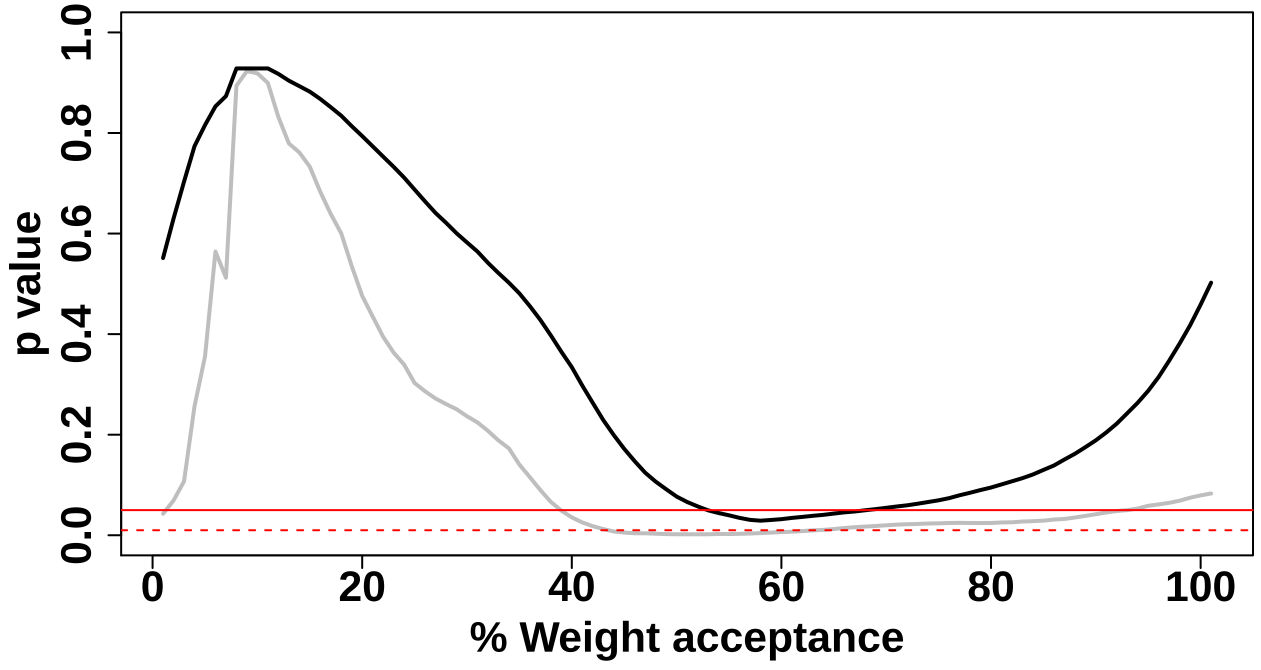

p value
0.0
0.2
0.4
0.6
0.8
1.0
0
20
40
60
80
100
% Weight acceptance

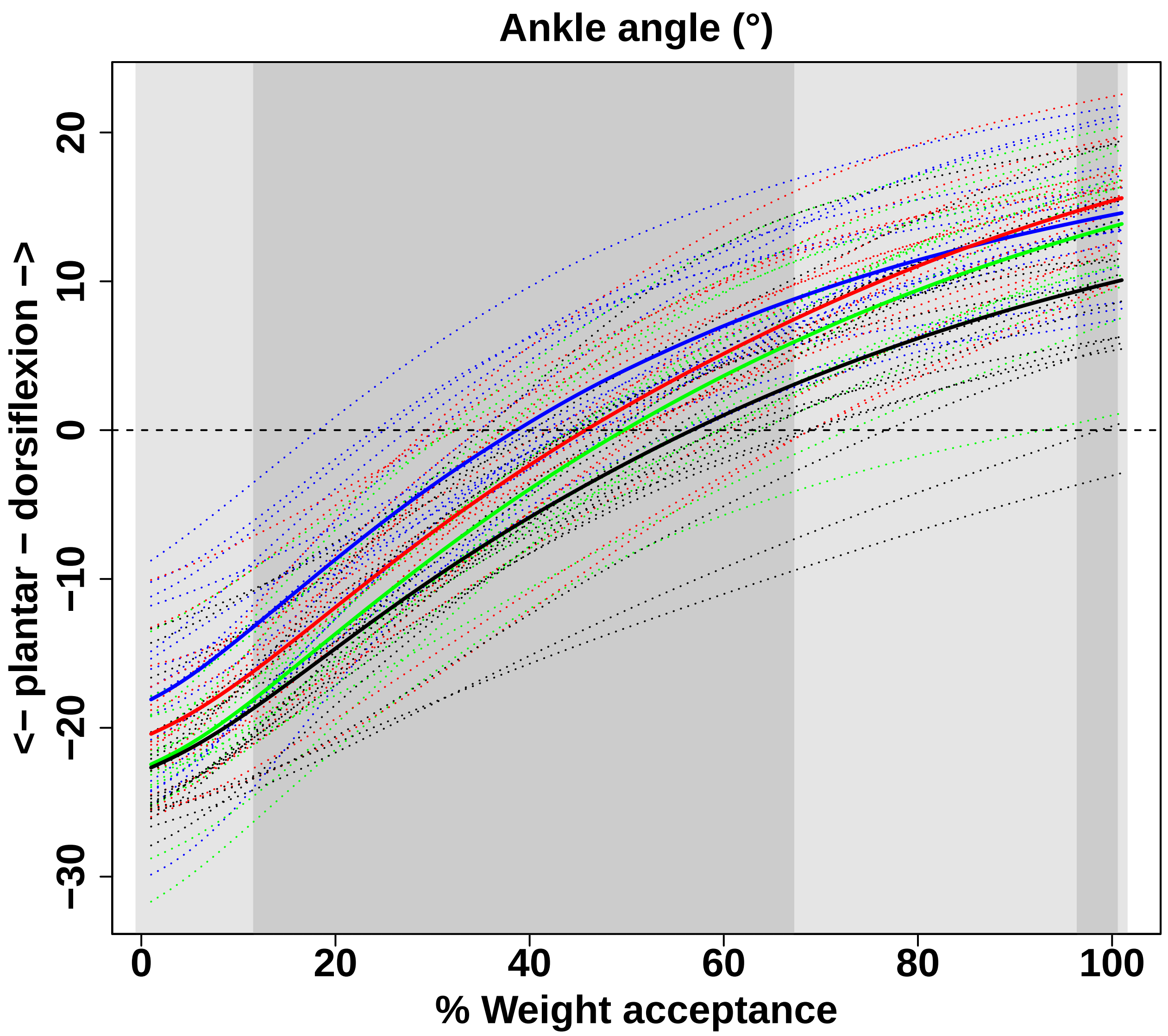

Ankle angle (°)
<– plantar – dorsiflexion –>
20
10
0
−10
−20
−30
0
20
40
60
80
100
% Weight acceptance

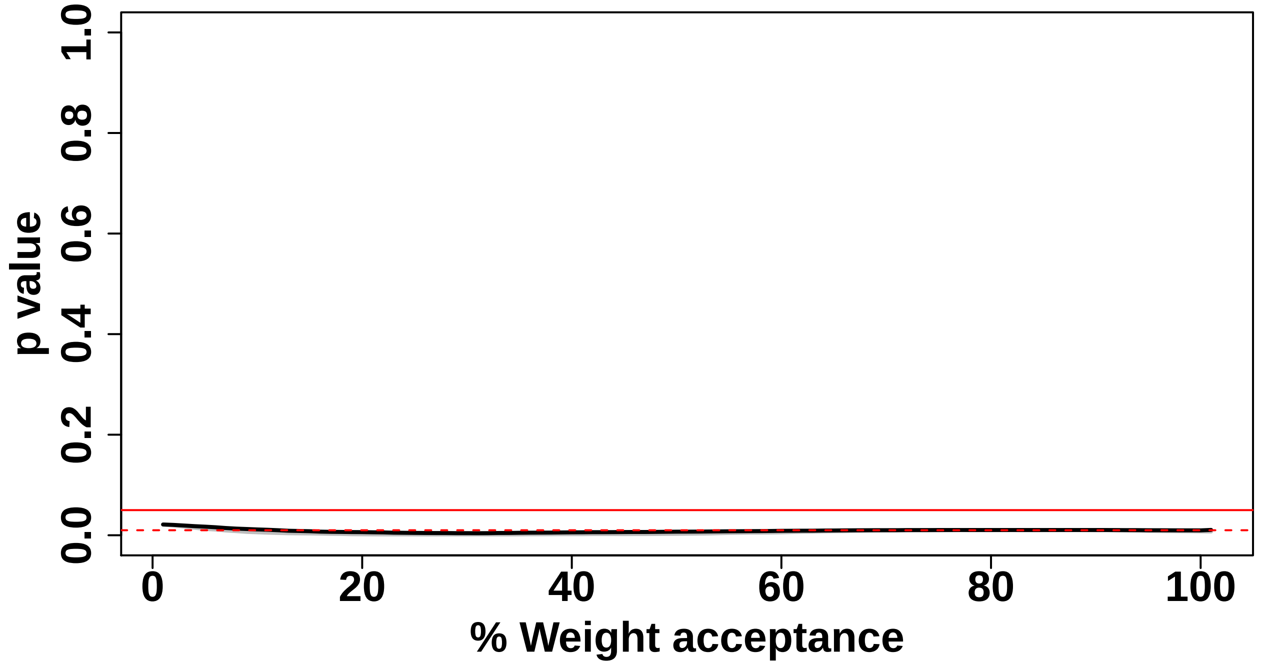

p value
1.0
0.8
0.6
0.4
0.2
0.0
0
20
40
60
80
100
% Weight acceptance

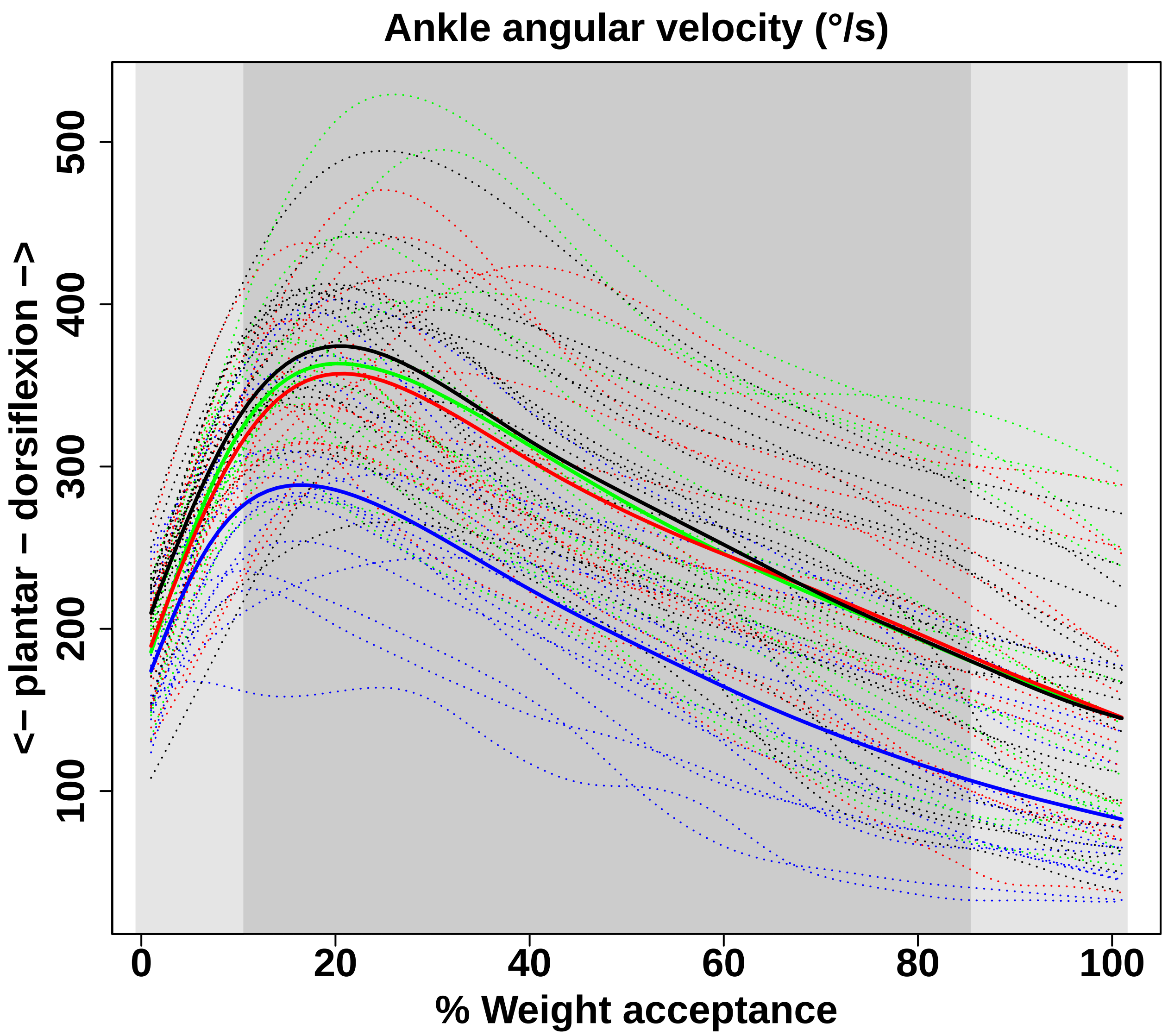
Ankle angular velocity (°/s)
<– plantar – dorsiflexion –>
500
400
300
200
100
0
20
40
60
80
100
% Weight acceptance

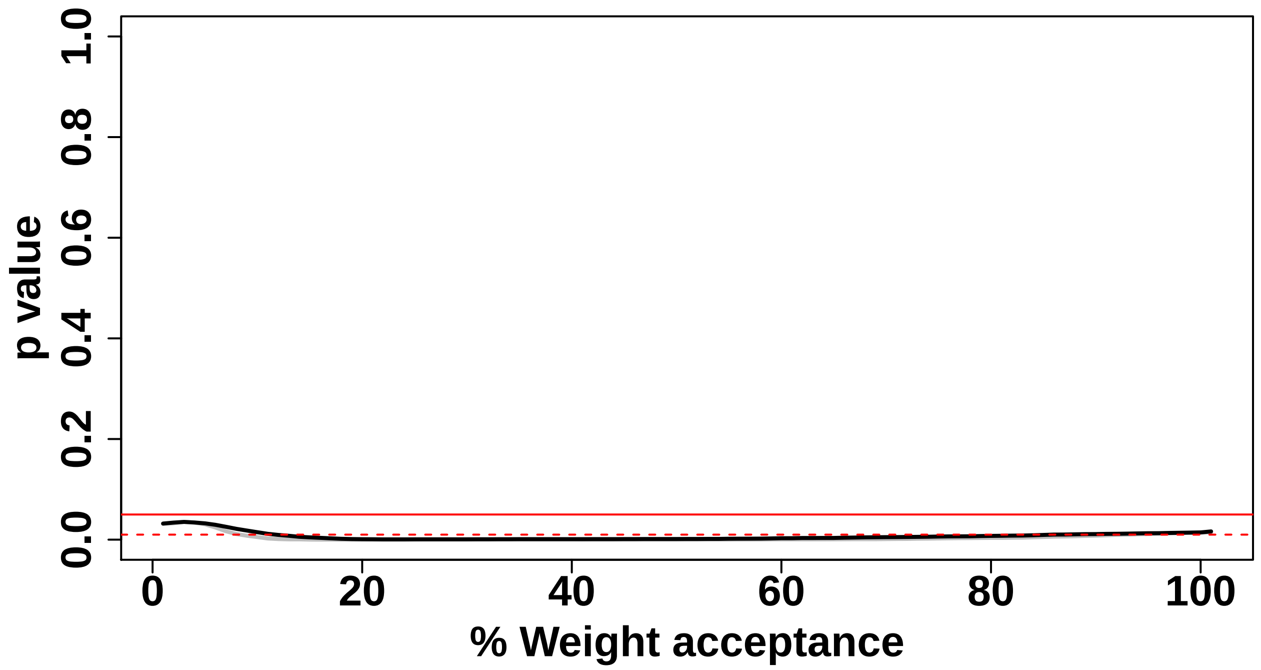
p value
1.0
0.8
0.6
0.4
0.2
0.0
0
20
40
60
80
100
% Weight acceptance

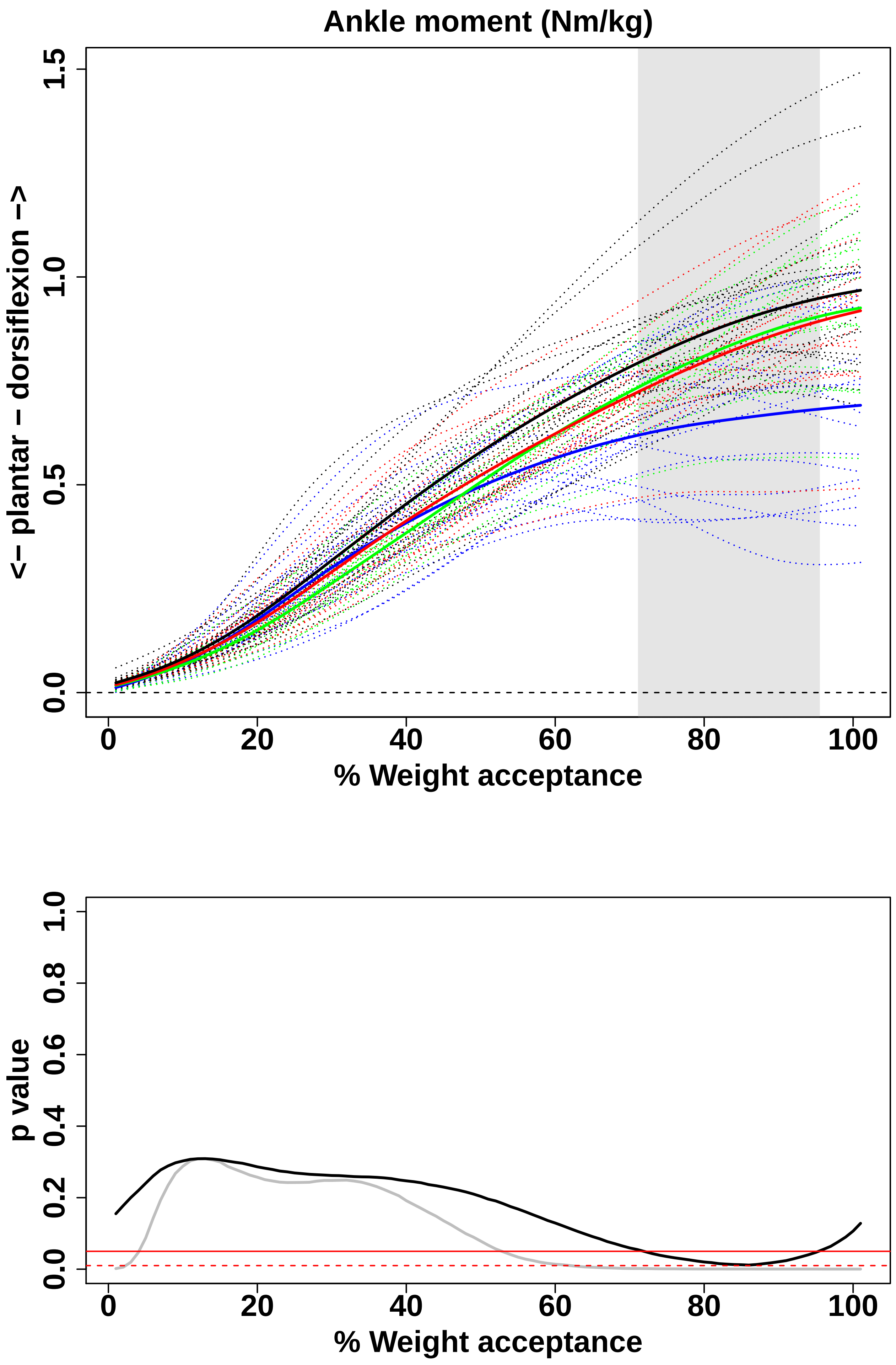

Ankle moment (Nm/kg)
<– plantar – dorsiflexion –>
1.5
1.0
0.5
0.0
0
20
40
60
80
100
% Weight acceptance
p value
1.0
0.8
0.6
0.4
0.2
0.0
0
20
40
60
80
100
% Weight acceptance

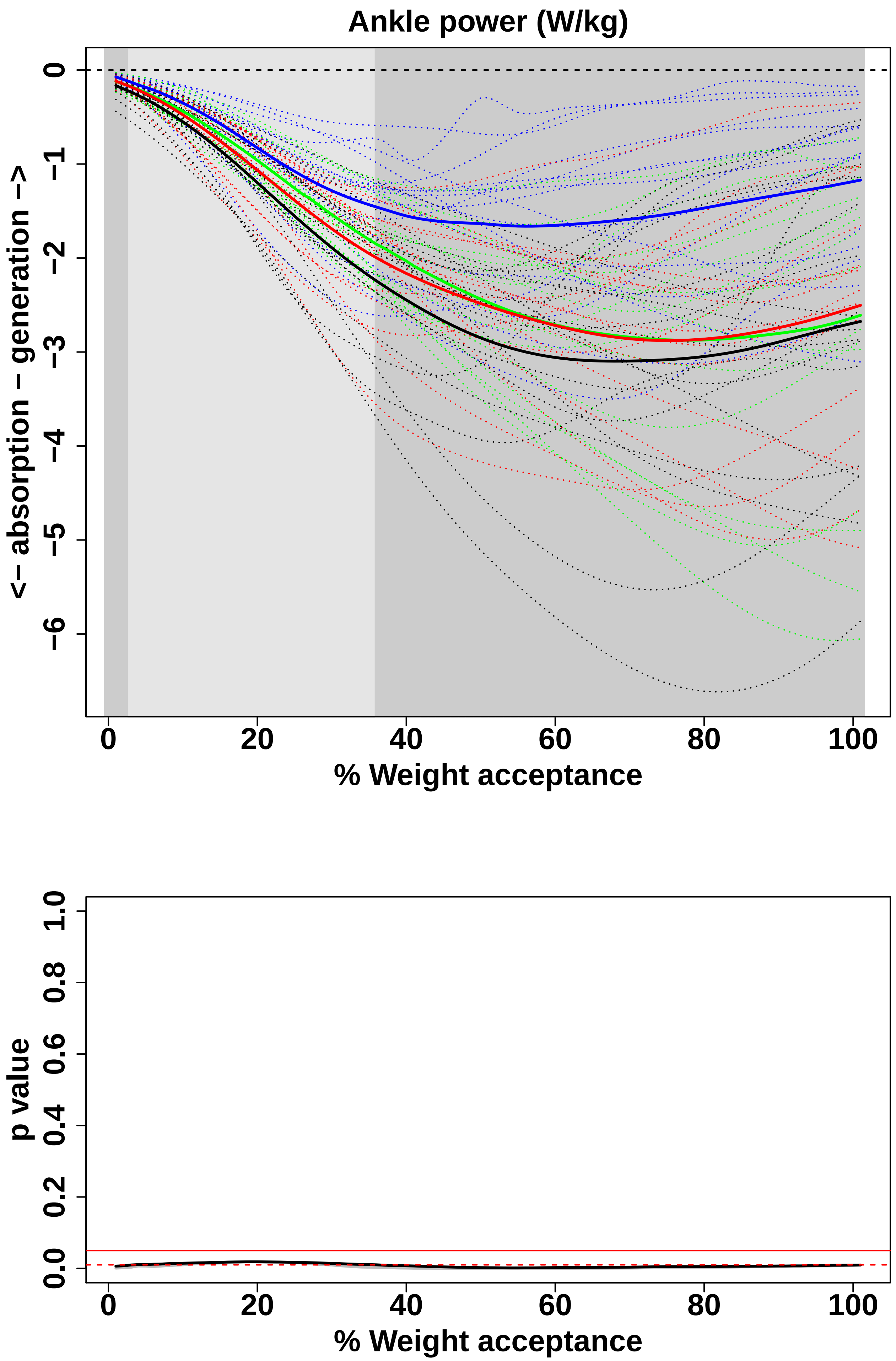

Ankle power (W/kg)
<– absorption – generation –>
0
−1
−2
−3
−4
−5
−6
0
20
40
60
80
100
% Weight acceptance
p value
0.0
0.2
0.4
0.6
0.8
1.0
0
20
40
60
80
100
% Weight acceptance
